# Caloric restriction mimetics: natural/physiological pharmacological autophagy inducers

**DOI:** 10.4161/auto.36413

**Published:** 2014-12-18

**Authors:** Guillermo Mariño, Federico Pietrocola, Frank Madeo, Guido Kroemer

**Affiliations:** 1 Equipe 11 labellisée par la Ligue Nationale contre le cancer; INSERM U1138; Center de Recherche des Cordeliers; Paris, France; 2 Université Paris Descartes; Sorbonne Paris Cité; Paris, France; 3 Université de Paris Sud; Villejuif, France; 4 Institute of Molecular Biosciences; University of Graz; Graz, Austria; 5 Metabolomics and Molecular Cell Biology Platforms; Gustave Roussy; Villejuif, France; 6 Pôle de Biologie; Hôpital Européen Georges Pompidou; AP-HP; Paris, France

**Keywords:** acetyl-coenzyme A, acetyl transferase, acetylation, deacetylase, deacetylation, AcCoA, acetyl coenzyme A, CRM, caloric restriction mimetics, EGCG, epigallocatechin-3-gallate

## Abstract

Nutrient depletion, which is one of the physiological triggers of autophagy, results in the depletion of intracellular acetyl coenzyme A (AcCoA) coupled to the deacetylation of cellular proteins. We surmise that there are 3 possibilities to mimic these effects, namely (i) the depletion of cytosolic AcCoA by interfering with its biosynthesis, (ii) the inhibition of acetyltransferases, which are enzymes that transfer acetyl groups from AcCoA to other molecules, mostly leucine residues in cellular proteins, or (iii) the stimulation of deacetylases, which catalyze the removal of acetyl groups from leucine residues. There are several examples of rather nontoxic natural compounds that act as AcCoA depleting agents (e.g., hydroxycitrate), acetyltransferase inhibitors (e.g., anacardic acid, curcumin, epigallocatechin-3-gallate, garcinol, spermidine) or deacetylase activators (e.g., nicotinamide, resveratrol), and that are highly efficient inducers of autophagy in vitro and in vivo, in rodents. Another common characteristic of these agents is their capacity to reduce aging-associated diseases and to confer protective responses against ischemia-induced organ damage. Hence, we classify them as “caloric restriction mimetics” (CRM). Here, we speculate that CRM may mediate their broad health-improving effects by triggering the same molecular pathways that usually are elicited by long-term caloric restriction or short-term starvation and that imply the induction of autophagy as an obligatory event conferring organismal, organ- or cytoprotection.

Macronutrient scarcity constitutes one the most common inducers of macroautophagy (to which we refer as autophagy). In teleological terms, the prime finality of autophagy is the mobilization of the cell's reserves and hence the conversion of macromolecules into energy-rich substrates that are required for maintaining essential functions, the avoidance of cell death, and the adaptation to stress.^1,2^ Starvation of human cells (by their culturing in nutrient-free medium) or starvation of mice (by removing food from the cages for 24 h, granting access only to water) results in the preponderant depletion of 1 intracellular metabolite, acetyl coenzyme A. Kinetic experiments performed in vitro, on human cell lines cultured in the absence of nutrients indicate that depletion of the nucleocytosolic pool of AcCoA occurs before ATP is reduced, NADH is oxidized, and amino acids are depleted from the intracellular metabolome, at the same time as autophagy becomes detectable.^3^ Specific depletion of cytosolic AcCoA pools by inhibition of its mitochondrial synthesis (from pyruvate, branched amino acids or lipid ß-oxidation) or its transfer from the mitochondrial matrix to the cytosol (which requires the conversion of AcCoA to citrate in the matrix, the export of citrate by the citrate carrier, and final conversion of citrate to AcCoA by ACLY [ATP citrate lyase]) is sufficient to induce autophagy even in conditions in which ATP and NADH levels are normal.^3^ Moreover, external provision of AcCoA (e.g., by microinjection of the metabolite into the cytoplasm) is sufficient to prevent starvation-induced autophagy.^3^

Altogether, these observations point to the idea that starvation causes autophagy because it results in the early depletion of AcCoA.^3,4^ This adds to other mechanisms through which caloric restriction or starvation can stimulate autophagy, namely the induction of the deacetylase activity of sirtuins (as a result of changing NADH/NAD^+^ ratios and increased SIRT1 expression),^5^ the activation of AMPK activity (as a result of changing ATP/ADP ratios),^6^ and the inhibition of MTORC1 (as a result of amino acid depletion).^7^ The available evidence indicates that the principal acetylransferase that is required for the AcCoA-mediated repression of autophagy is EP300,^3^ an acetyltransferase that can transfer acetyl groups from AcCoA to autophagy core proteins including ATG5, ATG7, ATG12, and LC3, thus inhibiting their pro-autophagic activity.^8^ Specific AcCoA depletion or direct inhibition of EP300 by genetic or pharmacological methods causes the rapid activation of AMPK and the inactivation of MTORC1, suggesting that these nutrient sensors are functionally connected to each other.^3^

The aforementioned results suggest a strategy for the identification of drugs that mimic the effects of starvation with regard to the depletion of AcCoA and the consequent deacetylation of cellular proteins. Within this framework, there would be 3 categories of “caloric restriction mimetics” (CRMs): (i) agents that reduce the concentration of cytosolic AcCoA; (ii) inhibitors of autophagy-repressive acetyltransferases including EP300; and (iii) activators of autophagy-stimulatory deacetylases including SIRT1.^9^ It is reasonable to expect that CRMs falling in one of these 3 categories would elicit the same biochemical pathways that are usually stimulated by starvation and hence induce an autophagic response that is exempt from major toxicological side effects (**Fig. 1**).

**Figure 1.**
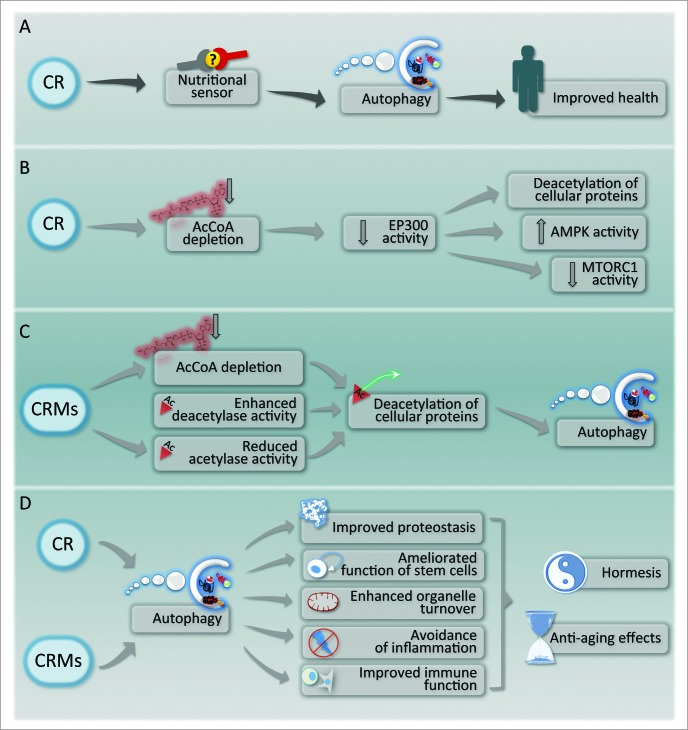
Caloric restriction and its pharmacological mimetics. (**A**) General outline of the mechanisms of health improvement by caloric restriction (CR). (**B**) Molecular mechanism of autophagy induction by CR. (**C**) Mechanism of action of caloric restriction mimetics (CRMs). (**D**) Hypothetical mechanisms of anti-aging effects of CR and CRMs.

Indeed there is a vast literature showing that there are multiple CRMs that can be used in humans. As an example, hydroxycitrate, an inhibitor of ACLY that causes cytosolic AcCoA depletion, protein deacetylation, and massive autophagy in all studied organs in mice,^3^ is also an over-the-counter weight loss agent commercialized in the US.^10^ A variety of agents known to inhibit EP300 are being used in traditional medicine or are obtainable without a prescription. This applies to anacardic acid (6-pentadecyl-salicylic acid from the nutshell of the cashew, *Anacardium occidentale*),^11^ curcumin (from the South Asian spice turmeric, *Curcuma longa*, one of the principal ingredients of curry powder),^12^ and garcinol (from the fruit of the Kokum tree, *Garcina indica*).^13^ All these agents are also potent inducers of protein deacetylation and autophagy when added to cultured human cells.^3^ Similarly, epigallocatechin-3-gallate (EGCG, one of the major active compounds contained in green tea) can inhibit a range of acetyltransferases^14^ including EP300.^15^ Spermidine (a polyamine contained in all organisms, but found at particular high concentrations in some health-related products such as durian fruit, fermented soybeans, and wheat germs) was first characterized as a histone acetyl transferase inhibitor.^16^ Spermidine potently induces protein deacetylation and autophagy in vivo, in mice or in cultured human cells.^17^ Finally, resveratrol exemplifies a widely used over-the-counter drug that can stimulate the deacetylase activity of SIRT1, thereby causing general protein deacetylation and autophagy.^17-19^ Nicotinamide is another potential SIRT1 activator that is sold over the counter in the US^20^ and that induces autophagy in rodents.^21^

What could be the therapeutic indications for the use of such CRMs? CR or intermittent fasting are known for their wide life-span-extending and health-improving effects that can be measured in an objective fashion in multiple model organisms including rodents^22^ and primates.^23^ Beyond their capacity to reduce aging and aging-associated pathologies (such as neurodegeneration, type-2 diabetes, and cancer), fasting also has an important preconditioning effect, protecting different organs from ischemic insult. This applies to the heart^24,25^ brain,^26^ liver,^27^ and kidney.^28^ There is emerging evidence that autophagy is involved in starvation-mediated organ protection.^25,28^ Moreover, fasting can reduce the subjective and objective toxicity of cytotoxic anticancer chemotherapies, both in humans and in mouse models, at the same time that it improves treatment outcome in mice.^29,30^ It is tempting to speculate that CRMs could be used for the same therapeutic indications in which fasting has proven to be useful. In accord with this idea, several CRMs can increase the health span and life span of rodents (as demonstrated for EGCG, spermidine and resveratrol),^31-33^ reducing the advancement of neurodegenerative diseases (as shown for spermidine, nicotinamide and resveratrol), likely through their capacity to induce autophagy.^34,35^ Moreover, several CRMs (including EGCG and resveratrol) have potent preconditioning effects in ischemia,^36,37^ which, at least on theoretical grounds, might be due to the induction of cytoprotective autophagy.^38^

Future studies should address the following major questions:

- Do all beneficial effects of CRMs result from the induction of autophagy, or are there any autophagy-independent effects? This question should be addressed in suitable mouse models in which autophagy can be genetically inhibited in a spatially- and temporarily-controlled fashion.
- Which are the CRMs that are optimally suitable for a precise indication (anti-aging effects, neuro-, cardio-, hepatoprotection, adjuvant treatment of anticancer chermotherapy), comparing them in preclinical tests to their positive control, that is fasting or caloric restriction? Ideally, this problem should be addressed in a systematic fashion involving the simultaneous comparison of multiple CRMs.
- Is it possible to develop more specific CRMs, such as inhibitors of EP300 that fail to affect other acetyltransferases or truly specific inhibitors of ACLY, with the scope of optimizing their efficacy?
- Is it possible to combine several mechanistically distinct CRMs (such as those depleting AcCoA, inhibiting acetyltransferases, or activating deacetylases) to obtain synergistic effects for maximal induction of therapy-relevant autophagy?

We surmise that responding to these questions will boost the rational development of new indications for old drugs, as well as the development of novel CRMs with a broad therapeutic potential.
